# Test your knowledge and understanding

**Published:** 2014

This page is designed to help you test your own understanding of the concepts covered in this issue and reflect on what you have learnt. We hope that you will also discuss these questions with your colleagues and other members of the eye care team, perhaps in a journal club. To complete the activities online – and get instant feedback – please visit www.cehjournal.org

**Table:** 

**1.**	**Good team work in eye care requires:**	True	False
**a.**	Communication pathways, both among personnel and between the different levels of service	□	□
**b.**	A clearly defined hierarchy	□	□
**c.**	Protocols to support the team, e.g. referral protocols	□	□
**d.**	An incentives and promotions structure	□	□
**2.**	**Leadership of the eye team means:**	True	False
**a**	Setting a direction and vision for the team, and fostering the right culture and values	□	□
**b**	Problem solving using existing systems and procedures	□	□
**c**	The same as management of an eye team	□	□
**d**	Motivating people by identifying opportunities for them to learn and practise new skills	□	□

## ANSWERS

**1.a. True.** This can be done through regular direct communication (including meetings) with the team, compiling lists of useful contacts and having SMS update systems between the different levels of service delivery. Communication is essential to keep the information flowing between team members. **b. False.** A hierarchy can have inherent power imbalances. People who feel that they have less power may be unwilling or unable to communicate, which makes teamwork difficult. **c. True.** A protocol is a standardised guide that will ensure that quality of services is reproducible. **d. False.** Incentives and promotions provide individuals with a career pathway, but cannot be regarded as central to motivating team work. For example, feedback on cataract outcome monitoring will motivate and encourage the team whereas a training workshop for a surgeon may only support her or his career.
**2.a. True.** In leading a team, the focus is on inspiring people and ensuring that the attention is on the bigger picture, whereas managing a team will focus on planning, budgeting and setting up management systems. Good leaders foster clear values and set a mission for a programme. **b. False.** A manager is focused on procedure-driven activities, e.g. accounts or protocols, whereas a leader with be focused on the outcome and impact of service delivery. **c. False.** Leaders have their eye on the vision and direction of the team; i.e., leaders are responsible for developing the team. Managers are focused on ensuring that each day functions well; i.e. managers maintain a team. Leaders have a role in ensuring that teams have the practical support they need, but are supported by managers in order to achieve this. **d. True.** A leader focuses on the people in the team and how they should/could be developed to enhance its functioning. It requires innovative approaches and motivation to do the right things.

